# ZnS@ZIF-8 core-shell nanoparticles incorporated with ICG and TPZ to enable H_2_S-amplified synergistic therapy

**DOI:** 10.7150/thno.45079

**Published:** 2020-06-18

**Authors:** Chao Fang, Dong Cen, Yifan Wang, Yongjun Wu, Xiujun Cai, Xiang Li, Gaorong Han

**Affiliations:** 1 State Key Laboratory of Silicon Materials, School of Materials Science and Engineering, Zhejiang University, Hangzhou 310027, P.R. China.; 2 Key Laboratory of Endoscopic Technique Research of Zhejiang Province, Sir Run Shaw Hospital, Zhejiang University, Hangzhou 310016, P. R. China.; 3 Hangzhou Global Scientific and Technological Innovation Center, Zhejiang University, Hangzhou, P.R. China.

**Keywords:** hydrogen sulfide, core-shell nanoparticles, indocyanine green, tirapazamine, synergistic therapy

## Abstract

Abnormal tumor microenvironment, such as hypoxia, interstitial hypertension and low pH, leads to unexpected resistance for current tumor treatment. The development of versatile drug delivery systems which present responsive characteristics to tumor microenvironment (TME) has been extensively carried out, but remains challenging. In this study, zeolitic imidazolate framework-8 (ZIF-8) coated ZnS nanoparticles have been designed and prepared for co-delivery of ICG/TPZ molecules, denoted as ZSZIT, for H_2_S-amplified synergistic therapy.

**Methods:** The ZSZ nanoparticles were characterized using SEM, TEM and XRD. The* in vitro* viabilities of cancer cells cultured with ZSZIT under normoxia/hypoxia conditions were evaluated by cell counting kit-8 (CCK-8) assay. In addition, *in vivo* anti-tumor effect was also performed using male Balb/c nude mice as animal model.

**Results:** ZSZIT shows cascade PDT and hypoxia-activated chemotherapeutic effect under an 808nm NIR irradiation. Meanwhile, ZSZIT degrades under tumor acidic environment, and H_2_S produced by ZnS cores could inhibit the expression of catalase, which subsequently favors the hypoxia and antitumor effect of TPZ drug. Both* in vitro* and *in vivo* studies demonstrate the H_2_S-sensitized synergistic antitumor effect based on cascade PDT/chemotherapy.

**Conclusion:** This cascade H_2_S-sensitized synergistic nanoplatform has enabled more effective and lasting anticancer treatment.

## Introduction

Photodynamic therapy (PDT), an oxygen-dependent therapeutic strategy, is driven by the induction of reactive oxygen species (ROS) from a photosensitizer under light irradiation 1. The efficacy of PDT suffers daunting challenges due to the intrinsic hypoxia environment of tumor tissue 2. A variety of strategies have been devoted to tackle this challenge, including direct supply of oxygen during PDT 3-6 or combining other oxygen-independent therapies to promote antitumor outcomes 7-10. Alternatively, the use of a hypoxia-activated prodrug, tirapazamine (TPZ), enables high toxicity to malignant tumor cells with intrinsic hypoxia, while showing negligible inhibition to normoxia cells 11, 12. However, the highly-toxic free radicals produced by TPZ molecules are likely to be scavenged rapidly by the uneven distribution of oxygen, hindering its expected therapeutic efficacy 13. The reaction during PDT consumes considerable content of oxygen, and thus aggravates the hypoxia tumor microenvironment (TME) which may significantly facilitate TPZ prodrug to induce cytotoxicity. It is therefore logic to combine PDT and TPZ-activated chemotherapy as a potential approach for synergistic therapy 14, 15. Meanwhile, recent studies revealed that the hypoxia condition could be relieved by highly-expressed catalase (CAT) in cancer cells which can transform intracellular H_2_O_2_ into oxygen 16-18. Thus, the toxic radicals produced by TPZ can be potentially oxidized and reversed back to nontoxic compounds, weakening its anticancer efficacy. The effective suppression of CAT within cancer cells has therefore been anticipated as a potential methodology to promote the efficacy of hypoxia-triggered therapeutics.

Treatment using specific gases has recently emerged as an attractive approach for antitumor purposes. A variety of gas molecules may regulate particular cellular pathways and in turn influence cell proliferation, apoptosis/death, metabolism, and so on 19-21. Similar to carbon monoxide (CO) and nitric oxide (NO), hydrogen sulfide (H_2_S), as an endogenous gaseous transmitter, is known to play a crucial role in physiological and pathophysiological processes 22-25. Several key proteins corresponding to cellular pathways are likely to be sulfhydrated and form protein persulfides by H_2_S, so-called S-sulfhydration 26, 27. It has been revealed that H_2_S may influence the survival and death of tumor cells in a double-edged manner 19, 28, 29. The presence of H_2_S, above a certain concentration, induces selective inhibition of tumor cells due to the distinctive metabolism and signaling pathways of tumor cells 22, 27. More interestingly, H_2_S could regulate the expression of CAT depending on cell types 30-32. Since CAT plays an important role in metabolism and redox balance of cancer cells 16, H_2_S is expected to be a potential gaseous inhibitor to fulfill the needs of CAT suppression in specific therapeutic applications.

**Figure 1:**
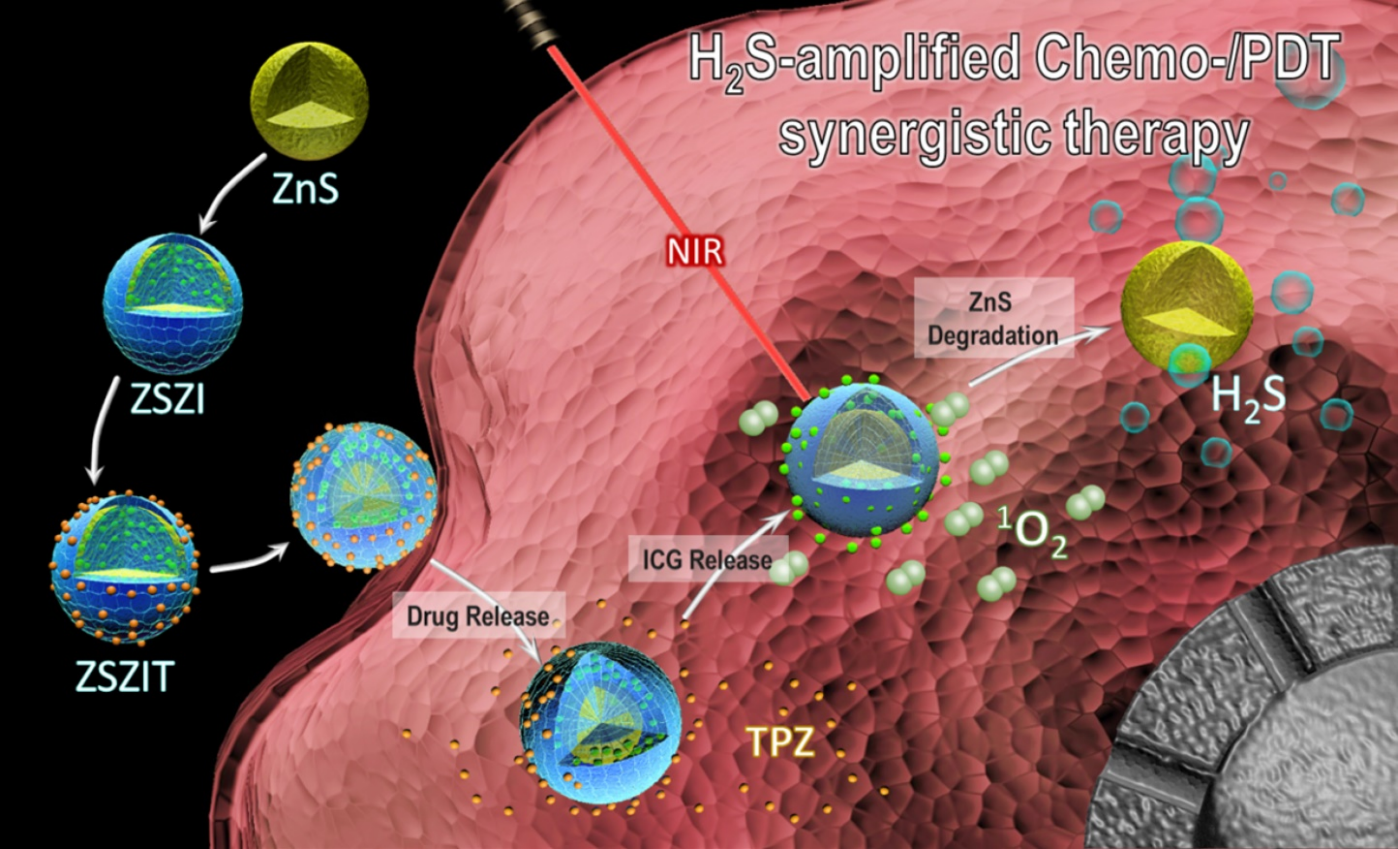
Schematic illustration of ZSZIT as a H_2_S-sensitized PDT/chemotherapeutic synergistic nanoplatform.

Herein in this study, fine core-shell nanoparticles, consisting of ZnS nanocrytals covered by zeolitic imidazolate framework-8 (ZIF-8) shell, were synthesized and incorporated with indocyanine green (ICG) and tirapazamine (TPZ), denoted as ZnS@ZIF-8/ICG/TPZ (ZSZIT), to enable H_2_S-sensitized PDT/chemotherapy synergistic therapy. As demonstrated in Figure 1, ZnS core, prepared *via* one-pot hydrothermal method, is coated with ZIF-8 by heterogeneous nucleation/growth process (ZSZ). ICG molecules are incorporated into the ZIF-8 shell during the coating procedure (defined as ZSZI), and subsequently TPZ is grafted on the surface of core-shell nanoparticles (ZSZI) to form ZSZIT. ZIF-8 shell here acts as a pH-responsive delivery cargo for ICG and TPZ. Owing to the protonation of imidazole in acidic tumor site, ZIF-8 shell collapses and consequently both ICG and TPZ release from the particles 33-35. 808 nm light source is used to trigger the ROS induction of ICG molecules released, and meanwhile consume the on-site oxygen. More importantly, in an acidic condition, ZIF-8 shell decomposes, and in turn ZnS core degrades to produce H_2_S gas *in situ*. The intracellular H_2_S does not only exhibit certain cytotoxicity, but also suppresses the expression of CAT which blocks the transformation pathway from H_2_O_2_ to oxygen, favoring the hypoxia condition in cancer cells. The considerable hypoxia condition induced by the reaction of ROS production as well as the suppressed CAT activity by H_2_S, aggravates the hypoxia of TME and agitates the cytotoxicity of TPZ molecules. Consequently, significant tumor inhibition is achieved, both *in vitro* and *in vivo*, due to the combined effects of ROS, H_2_S and hypoxia-activated TPZ enabled by ZSZIT nanocomposites, indicating its considerable potential for effective cancer treatment.

## Materials and Methods

### Materials

Zinc acetate (99%), thiourea (AR, 99%), indocyanine green (ICG), iron (III) chloride (FeCl_3_) and 1,3-diphenylisobenzofuran (DPBF) were purchased from Aladdin Co. Ltd. Polyvinylpyrrolidone (K30), Zinc nitrate hexahydrate (AR), methanol anhydrous, sulfuric acid and sodium acetate anhydrous were obtained from Sinopharm Chemical Reagent Co. Ltd. Tirapazamine and CCK-8 were supplied by Dalian Meilun Biotechnology Co. Ltd. Poly (sodium 4-styrenesulfonate) (PSS, Mw = ~70000), 2-methylimidazole, N, N-Dimethyl-p-phenylenediamine (≥99.0%) and dichlorofluorescein diacetate (DCFH-DA, ≥97%) were purchased from Sigma-Aldrich Co. Ltd.

### Characterization

The microstructure of nanoparticles was examined by field-emission scanning electron microscopy (FESEM, Hitachi SU-70) and transmission electron microscopy (TEM, Tecnai F20, FEI). The crystal structure was characterized by X-ray diffraction with Cu Kα radiation (XRD, X'pert PRO MPD). Zeta potential of nanoparticles in extra-pure water (pH ~ 7) was determined by Zetasizer (Zetasizer Nano-ZS, Malvern). The Fourier transform infrared spectroscopy (FTIR) spectra were recorded using a PerkinElmer 580B (Tensor 27, Bruker). The UV-vis adsorption was characterized by a UV-vis spectrophotometer (UV2600, Shimadzu).

### Synthesis procedure

ZnS nanoparticles were synthesized *via* a modified hydrothermal method according to the reported literature 36. Briefly, 0.8 mmol (0.14678 g) zinc acetate was dissolved in 20 mL deionized water (DI), and 1 g PVP (K30) and 20 mmol (1.5224 g) thiourea were added in sequence. After stirring for 10 min, the transparent solution was transferred to a 50 mL stainless Teflon-lined autoclave and maintained at 140 °C for 50 min. The as-prepared ZnS nanoparticles were collected by 12000 rpm centrifugation for 10 min.

ZnS@ZIF-8 (ZSZ) nanoparticles were synthesized as follows: 5.4 mg ZnS nanoparticles were dispersed in 0.3wt% PSS aqueous solution to modify the nanoparticle surface with anionic surfactant by sonication for 90 min. After 12000 rpm centrifugation, the nanoparticles were transferred to a glass beaker containing 12 mL methanol mixed with 40 mg PVP (K30). After stirring, 2 mL Zn(NO_3_)_2_ (0.043 M) methanol solution was added into the mixture followed by adding 8 mL 2-methylimidazole (0.043 M) methanol solution dropwise. ICG loading was performed during the ZIF-8 coating procedure, with a certain concentration of ICG added into the mixture before adding 2-MIM dropwise to form uniform ZIF-8 coating. After 6 h stirring, nanoparticles were collected by centrifugation and washed with methanol for three times. Then, ZnS@ZIF-8/ICG (ZSZI) was dispersed in deionized water for subsequent TPZ loading 37. Briefly, 5 mg TPZ was added into above aqueous solution and stirred for another 10 h. After centrifugation, the samples were collected and washed for further use.

The drug loading capacity and encapsulated efficiency of ZnS@ZIF-8/ICG (ZSZI) were determined by UV-vis spectrophotometer. After 6 h of ICG loading, supernatant was collected by centrifugation, and the concentration was determined by standard curve. The drug loading capacity (LC) was calculated according to the following formula: Loading Capacity (%) = (The loaded drug mass)/(The loaded drug mass + The total mass of nanoparticles) × 100%; Meanwhile, the encapsulation efficiency (EE) was calculated as follows: Encapsulation Efficiency (%) = (The loaded drug mass)/(The total drug mass) × 100%. The calculation of loading capacity and encapsulated efficiency of TPZ is similar to the ICG.

The drug release properties of ICG and TPZ were also determined by UV-vis spectrophotometer. Samples were dispersed in buffer solution (4 mL in all) with different pH (4.7, 5.8 and 7.4) under gentle shaking at 37 °C. Subsequently, 2 mL supernatant was collected after 12000 rpm centrifugation for 10 min and another 2 mL fresh buffer solution was added. At various time periods (0, 1, 2, 4, 8, 12, 24 and 48 h), supernatant was collected for further measuring. The concentration of ICG/TPZ was calculated using a standard curve.

### Extracellular photodynamic property

The photodynamic property was measured using an ROS probe, diphenylbenzofuran (DPBF). Briefly, 100 μL DPBF solution (2.5 mM) was added into 3 mL of ZSZI solution (100 μg/mL) and irradiated with 808 nm NIR laser (1.0 W/cm^2^) for different time periods (0, 1, 2, 4, 8 and 16 min). Then the absorption was measured by UV-vis spectrophotometer at characteristic peak of 410 nm. The degradation of DPBF in dark was also detected following the same procedure.

### Exploration of pH-triggered degradation and H_2_S release properties

For H_2_S release property, ZSZ nanoparticles (200 μg/mL) were dispersed in buffer solution (8 mL in all) with different pH (4.7, 5.8 and 7.4) under gentle shaking at 37 °C. Subsequently, 4 mL supernatant was collected after 12000 rpm centrifugation for 10 min and another 4 mL fresh buffer solution was added. At various time periods (0, 2, 4, 8, 12, 24 and 36 h), supernatant was collected for further measuring. The concentration of H_2_S was measured using a standard method according to our previous study 38. Briefly, 1 mL of solution was mixed with zinc acetate/sodium acetate mixture (4:1 mass ratio, 1 mL). Methylene blue (MB) was then formed by the addition of N, N-dimethyl-p-phenylenediamine dihydrochloride (DMPD, 0.08 g/56 mL, 0.5 mL) and FeCl_3_ (0.2 g/40 mL, 0.5 mL). After incubation for 20 min, the absorbance at 665 nm was examined, and the concentration of H_2_S was determined using a standard curve of Na_2_S solution. Furthermore, TEM and UV-vis spectrophotometer were used to examine the degradation of ZSZ nanoparticles (Concentration: 20 μg/mL).

### *In vitro* study

7702 normal cells and Huh7 cancer cells were used for the *in vitro* study. Cells were cultured with DMEM culture medium containing 10% fetal bovine serum and 1% penicillin and streptomycin in a 37 °C incubator with 5% CO_2_. Cells were seeded in 96-well culture plates at a density of 20000 cells per well and incubated at 37 °C for 12 h before use. The medium was replaced with fresh DMEM medium containing ZSZI and ZSZIT at different concentrations. Normal and hypoxia conditions were provided using incubators with 20% and 2% oxygen atmosphere, respectively. The cytotoxicity of ZSZI and ZSZIT was also measured at different pH conditions. After 24 h incubation, 10 μL CCK-8 was added. After incubation for further 1 h, the absorbance at 450 nm was recorded by microplate reader.

### *In vitro* H_2_S-amplified PDT/chemotherapy synergistic effect

Huh7 cells were seeded in 96-well culture plates at a density of 20000 cells per well and incubated at 37 °C for 12 h. The DMEM culture medium was removed, and fresh DMEM containing samples with various concentrations were added. Before NIR irradiation, the cells were cultured with samples for further 4 h. To examine the H_2_S-activated synergistic properties, cells were incubated with ZSZI and ZSZIT under different conditions and irradiated with 808 nm laser. To induce H_2_S release, the pH of DMEM was set at 6.0. Furthermore, cytotoxicity of ZSZI and ZSZIT was also examined under hypoxia condition to demonstrate the hypoxia-activated chemotherapy. The power density of laser light was set at 1.0 W/cm^2^. After incubation for 24 h, CCK-8 assay was used to quantify the cell viability.

Besides, the live&Dead was also carried out using Calcein AM/PI staining. After seeded in 6-well plate and cultured for 12 h, Huh7 cells were treated with PBS, ZSZIT+Dark, ZSZI+NIR, ZSZIT+NIR and ZSZIT+NIR (pH=6) for another 10 h, following by dying with Calcein AM and PI and observation at 480 nm and 525 nm respectively.

### Intracellular ROS detection

ROS production within Huh7 cells after NIR irradiation was detected using 2', 7'-dichlorodihydrofluorescein diacetate (DCFH-DA) which can be oxidized by ROS to form highly fluorescent DCF. Briefly, after cell incubation with ZSZI nanoparticles (80 μg/mL) in 6-well culture plates in dark for 5 h, and 10 μL fresh DCFH-DA solution (2 mg/mL) was added to per well. After incubated in dark for further 30 min, the cultured cells were irradiated with 808 nm laser for 3 min (1.0 W/cm^2^). The cells were then incubated in dark for 1 h in a 37 °C incubator. Subsequently, cells were fixed with 4% formaldehyde solution after washing with PBS. After 10 min, the cells were washed for three times with PBS. 500 μL of 4', 6-diamidino-2-phenylindole (DAPI, 1 μg/mL) solution was added to each well and maintained for 10 min. Finally, fluorescence images were obtained using an inverted fluorescent microscope (Excited wavelength: 485 nm).

### Intracellular H_2_S detection

Intracellular H_2_S was detected by washington state probe-1 (WSP-1). Briefly, after being treated with ZSZI nanoparticles (80 μg/mL) under different pH, Huh7 cells were added with WSP-1 (15 μM) and incubated for 30 min. The cells were observed using a fluorescent microscope after washing with PBS.

### Intracellular O_2_ evaluation

Intracellular oxygen was detected by [Ru(dpp)_3_]Cl_2_ (RDPP). Firstly, Huh7 cells were seeded in 6-well plates and incubated in 37 °C for 12h. The medium was replaced with fresh DMEM containing PBS or ZSZI nanoparticles for further 4 h (80 μg/mL), and then cells were treated in dark or with NIR irradiation (1.0 W/cm^2^, 3min). The medium was removed, and fresh medium containing 5 μM RDPP was added and co-cultured for 4 h. The RDPP fluorescence was observed at 480 nm using an inverted fluorescent microscope.

### Intracellular CAT activity evaluation

CAT activity was determined using Catalase Assay Kit. Typically, cells were treated with ZSZIT at pH=6 for 30 minutes (0, 40 and 80 μg/mL). The cells were collected and washed for 3 times. The later procedure was according to kit protocol, and relative CAT activity was calculated.

### *In vivo* study

All animal experiments were performed humanely in compliance with guidelines reviewed by the animal ethics committee of the Biological Resource Centre of the Agency for Science, Technology and Research, Zhejiang University. Male Balb/c nude mice (4-6 weeks old) were purchased from Shanghai Laboratory Animal Center. To create the tumor model, Huh7 cells (5 × 10^6^) in PBS (200 μL) were injected subcutaneously into the right side back of each mouse. Tumor volume (V) was calculated as V = (width^2 × length)/2. When the tumor volume reached ~100 mm^3^, mice were randomly divided into 6 groups with different treatments (7 mice/group, intratumoral injection): Control group with injection of PBS (Group 1), injection of ZSZI without NIR irradiation (Group 2), injection of ZSZI with NIR irradiation (Group 3), injection of ZSZIT without NIR irradiation (Group 4), injection of free ICG and TPZ mixture solution with NIR irradiation (Group 5) and injection of ZSZIT with NIR irradiation (Group 6). Injection of free ICG and TPZ was set equivalent with injection of ZSZIT. After 2 h injection when the effective cellular uptake occurred (Figure S1), the tumor area of Group 3, 5 and 6 was irradiated with 808 nm-NIR (1.0 W/cm^2^) for 3 min 8, 39. Mice body weight and tumor size were recorded every two days in following 14 days. The therapeutic effect was evaluated by measuring the tumor size. Tumor volume was calculated with the following equation: V = (width^2 × length)/2. The changes of tumor size were evaluated by comparing the relative tumor volume, (V/V_0_, where V_0_ is the initiate tumor volume before treatment). On day 14, all the mice were sacrificed, and tumors were collected and weighted.

After the 14-day treatment, the tumor tissues from the control group and treated mice were histologically examined. Six groups of tumors were collected and fixed in 4% paraformaldehyde solution embedded in paraffin using a routine method. H&E staining of the tumor samples was undertaken following standard H&E staining procedures and the tissue slices were observed using an inverted microscope system.

*In vivo* biodistribution and biosafety were evaluated. To explore the *in vivo* distribution, two groups of mice (Control and ZSZIT treated groups) were sacrificed after 1-day treatment. Main organs (heart, liver, spleen, lung and kidney) were collected. The content of Zn element remained in the organs was examined by inductively coupled plasma-mass spectrometry (ICP-MS) and calculated as Zn percentage over injected dose per gram of tissues. In addition, hematoxylin and eosin (H&E) staining was also carried out to study the biosafety the ZSZIT.

### Statistical analysis

All data in this article are expressed as mean ± SD, and all comparison results between experimental groups were calculated through Student's t-test. Variations in the data were considered to be significant when ***p < 0.001, **p < 0.01 or *p < 0.05.

## Results and Discussion

### Synthesis of ZnS@ZIF-8/ICG/TPZ (ZSZIT)

**Figure 2:**
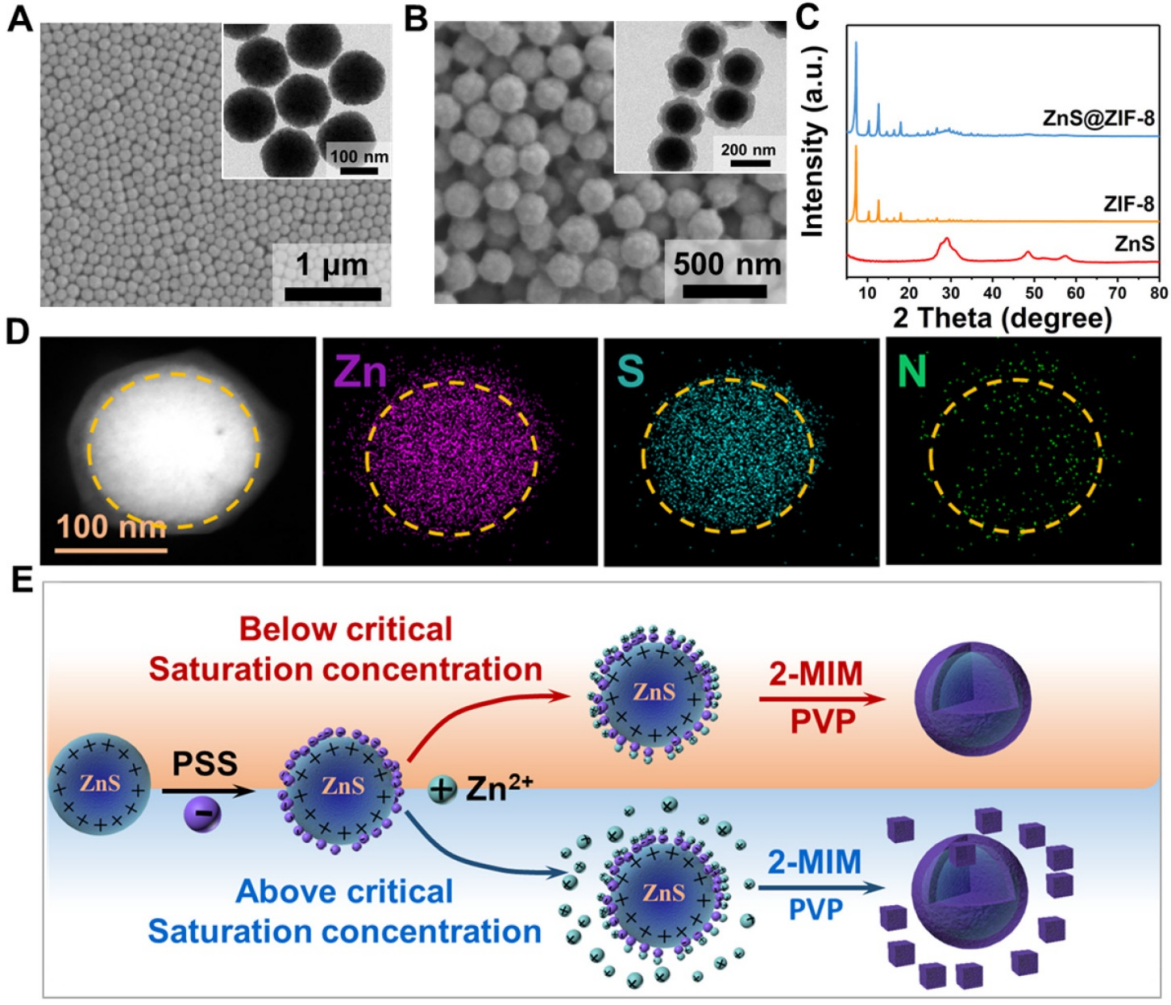
SEM images (Inset: TEM images) of (A) ZnS nanoparticles and (B) ZnS@ZIF-8 nanoparticles. (C) XRD pattern of ZnS and ZnS@ZIF-8 nanoparticles. (D) EDS element mapping of ZnS@ZIF-8 nanoparticles. (E) Schematic representation for the forming mechanisms of ZSZ under different conditions.

Uniform ZnS nanoparticles, with a spherical morphology and mean diameter of ~120 nm, were prepared prior to the coating procedure of ZIF-8 (Figure 2A). ZnS nanoparticles were then surface-modified with PSS molecules to attract Zn^2+^ ions, favoring the nucleation and growth of ZIF-8 shell at its surface. PVP may act as the stabilizer to prevent agglomeration and facilitates the formation of ZIF-8 shell 37, 40, 41. As shown in Figure 2B, the ZIF-8 shell, with a thickness of ~25 nm, is formed at the surface of ZnS cores. The particle diameter increases gently, and the surface roughens. It is worthnoting that the particle morphology does not present clear variation after PSS modification (Figure S2A). However, since the saturation concentration in aqueous solution is different comparing to methanol solution, a separated nucleation and growth of ZIF-8 particles occurs in aqueous solution (Figure S2B), while a uniform coverage of ZIF-8 is formed in methanol solution (Figure S2C). By increasing the Zn^2+^ concentration during the synthesis, the shell thickness can be feasibly increased to ~50 nm (Figure S3). The X-ray diffraction spectra show that ZSZ nanoparticles possess the characteristic peaks of both ZnS and ZIF-8 (Figure 2C). Elemental mapping verifies the unique core-shell structure of ZSZ nanoparticles prepared (Figure 2D and Figure S4). The sulfur element distributes in the core regime, indicating the core of ZSZ. Meanwhile, Zn and N are distributed throughout the whole particle including the ZIF-8 shell, as expected. The results of XPS analysis agree well to the elemental mapping (Figure S5). As demonstrated in Figure 2E, when the Zn^2+^ source reaches or excesses the critical saturation concentration, ZIF-8 flakes form separately in aqueous solution. In contrast, ZIF-8 coverage nucleates and grows, in a uniform manner, at the surface of ZnS nanoparticles below the saturation content in the methanol solution with a higher critical saturation concentration.

**Figure 3:**
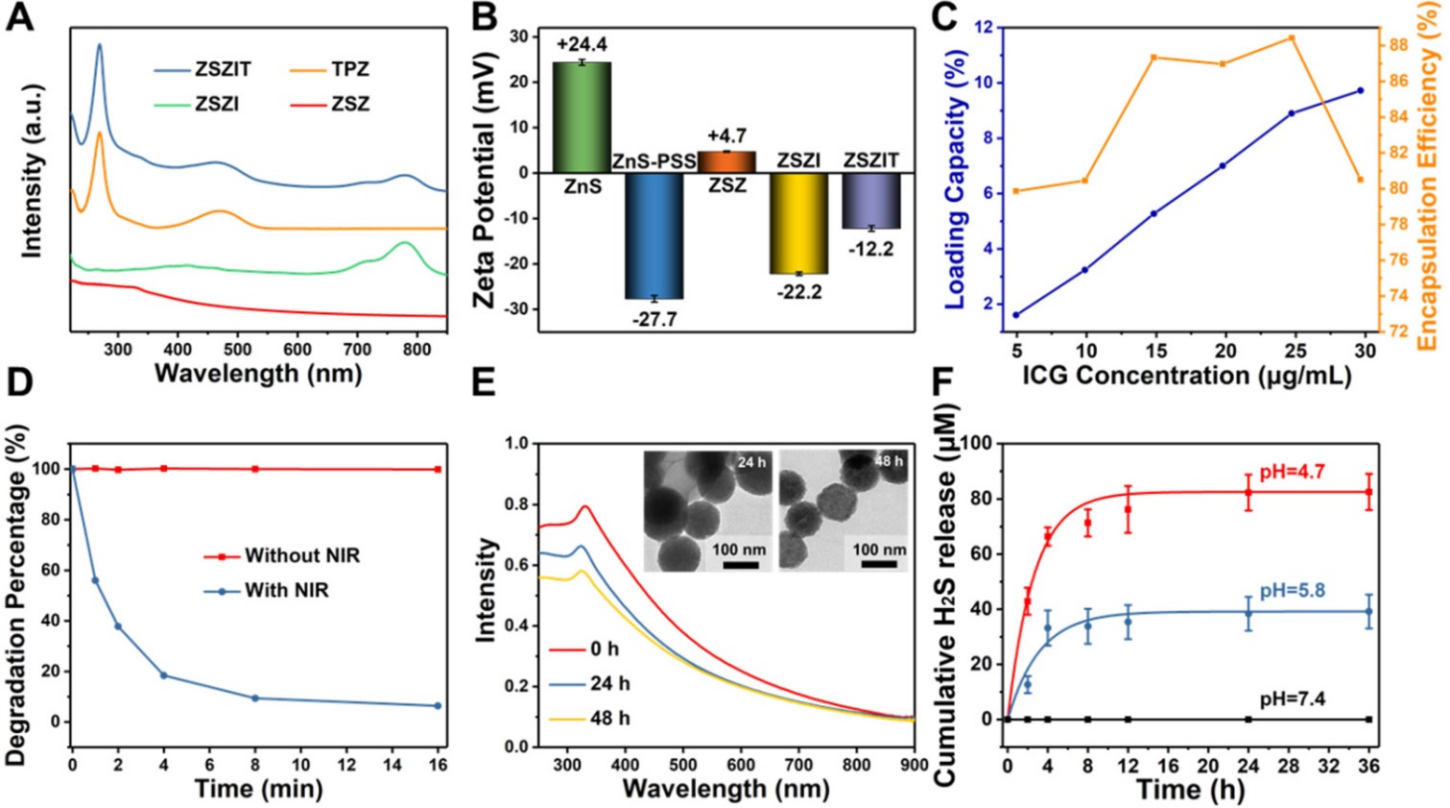
Surface modification and ICG/TPZ loading. (A) UV-vis absorbance spectra of ZSZ, ZSZI, TPZ and ZSZIT; (B) Zeta potential of ZnS, ZnS-PSS, ZSZ, ZSZI and ZSZIT in extra-pure water (pH ~ 7)); (C) Loading capacity and encapsulation efficiency of ICG during the synthesis. (D) DPBF degradation of ZSZI with/without 808 nm laser irradiation. (E) UV-vis spectra of ZSZ solutions at different time interval (pH = 5.8) (Inset: TEM images) and (F) H_2_S release behavior of ZSZ nanoparticles (200 µg/mL, ZnS content: ~71%).

ICG molecules were trapped in the shell of particles during ZIF-8 coating procedure (denoted as ZSZI, Table 1), and TPZ was then loaded onto ZSZI nanoparticles to form final nanocomposites, ZSZIT (Table 1). The incorporation of ICG and TPZ loading was examined using UV-vis spectrometry and Zeta potential (Figure 3A and 3B). Characteristic peaks at 780 nm and 268 nm present in ZSZIT sample, which are attributed to intrinsic absorption of ICG and TPZ molecules, respectively. Following the zeta potential examination, PSS modification changes the particle surface charge from positive to negative, and ZSZ exhibits positively charged surface due to the ZIF-8 coating. The variation of surface charge verifies the successful loading of ICG and TPZ, whose zeta potentials were -22.2 mV and -12.2 mV, respectively. Furthermore, FT-IR spectra of ZSZIT display the characteristic peak of ICG and TPZ (Figure S6). The variation of particle solution after loading ICG and TPZ can be feasibly observed by naked eyes due to its clear color changes (Figure S7). More ICG molecules could be incorporated into ZIF-8 shell with the increasing of loading concentration owing to its intrinsic high porosity. The encapsulation efficiency (EE) of ICG on the ZSZIT reaches the peak value of ~88.5% when the ICG concentration of 25 μg/mL was used during synthesis (Figure 3C and Figure S8). Accordingly, the loading capacity (LC) is ~8.6%, which is applied for the following experiments. The loading capacity and encapsulation efficiency of TPZ are 16.5% and 49.4%, respectively (Figure S9A and S9B). The TPZ release behavior has also been studied at different pH values (Figure S9C). Only ~20% of TPZ could be released after 4 h at pH=7.4. In comparison, ~60% of TPZ is released after 4 h at pH=5.8, indicating a pH-triggered TPZ releasing. When the pH of solution was decreased to 4.7, TPZ release accelerates, and completes in 4 h, which is of similar trend to ICG release (Figure S9D). The results of dynamic light scattering (DLS) indicate that ZSZIT nanoparticles maintain its stability in pure water, PBS and DMEM solutions for 12 h (Figure S10).

### Extracellular properties

The induction of ROS, singlet oxygen (^1^O_2_) in our study, by the core-shell particles under 808 nm NIR irradiation was examined using DPBF probe which presents intrinsic peak at ~410 nm for UV-vis analysis 8. DPBF is rapidly degraded in ZSZI solution under the 808 nm NIR irradiation, indicating its considerable properties in the ^1^O_2_ production. In comparison, DPBF hardly degrades under the same condition but without NIR irradiation (Figure 3D and S11).

The degradation phenomenon of core-shell structure and H_2_S release properties are crucial characteristics of nanoparticles for the antitumor purposes in this study. When incubated in pH = 5.8 buffer solution as mimicking acidic tumor microenvironment, nanoparticles present a clear degradation phenomenon. After 48 h, ZIF-8 shell collapses owing to the protonation of imidazole. As verified by the UV-vis spectra, the absorption continuously decreases after 24 h and 48 h incubation (Figure 3E), which can directly be visualized in the TEM images (Inset). Furthermore, the particle may degrade in a more rapid fashion when a more acidic solution (pH = 4.7) is used. In comparison, the particle could remain stable in a neutral condition (Figure S12). In general, ZIF-8 is a nontoxic and biocompatible zeolitic imidazolate framework (ZIF) constructed with zinc ions and 2-methylimidazolate 42. It remains stable in neutral water or aqueous sodium hydroxide, but decomposes quickly in acid solution, enabling an excellent TME-responsive characteristic 43. More specifically, ZIF-8 could respond to acidic conditions with pH of 5.0-6.5, and avoid potential premature drug release in normal tissues 44. More interestingly, after the collapse of ZIF-8 shell, a clear H_2_S release is detected. H_2_S was released from ZSZ solution continuously when the pH was set at 5.8, and the increased acidity accelerated the H_2_S release kinetics of core-shell nanoparticles, which was similar to the bare ZnS nanoparticles (Figure 3F, Figure S13 and Figure S14). As shown in Figure 3F, no clear sign of H_2_S release was observed under a neutral condition (pH=7.4). In comparison, the cumulative H_2_S release in solutions at pH = 5.8 and pH = 4.7 after 24 h reached ~38.4 μM and ~82.3 μM, respectively. The pH-dependent H_2_S release is ascribed to the ionization of ZnS cores in acidic condition, which could provide sustainable H_2_S release in tumor microenvironment.

### *In vitro* study

**Figure 4:**
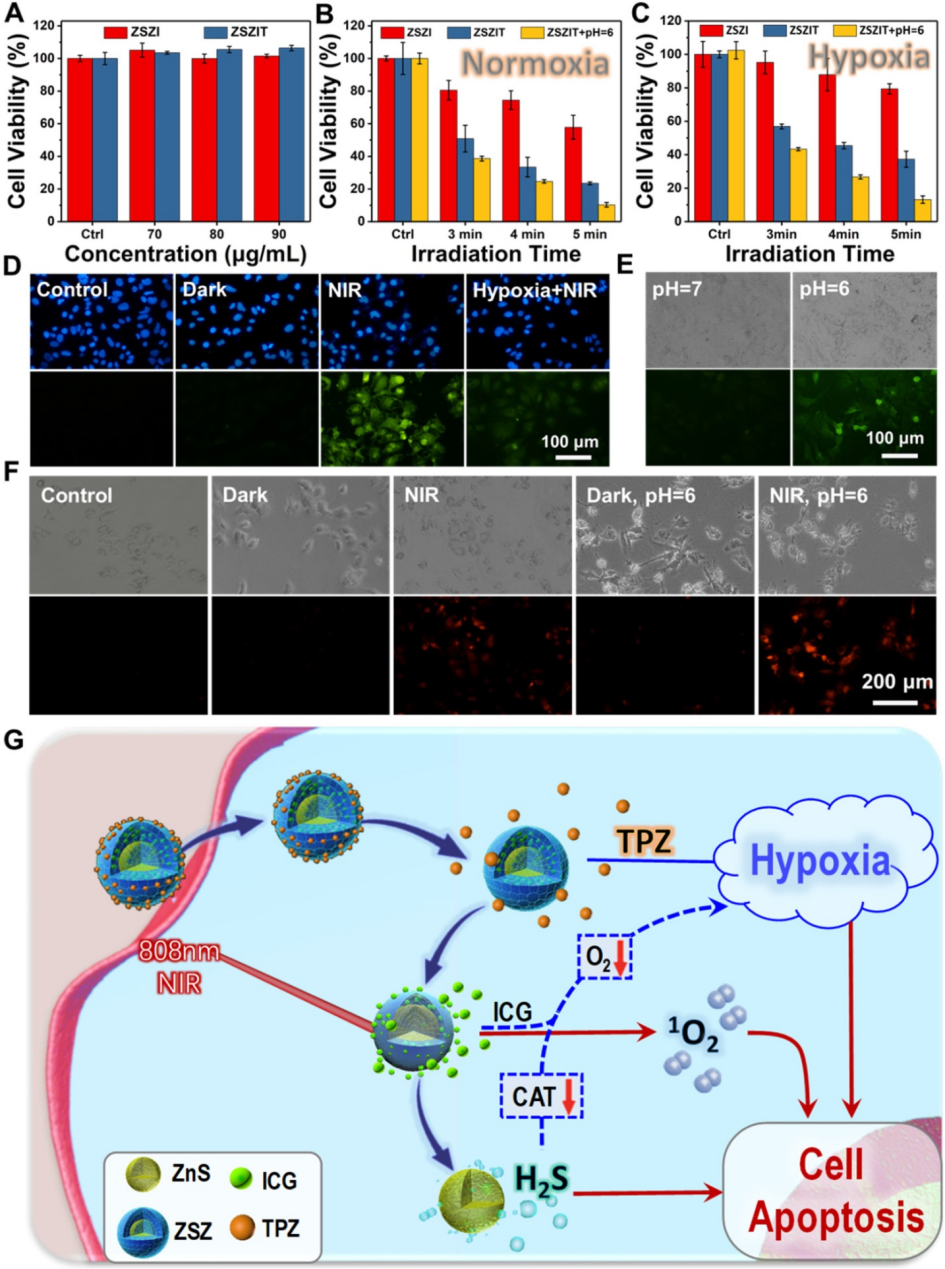
(A) Huh7 cell viabilities of ZSZI and ZSZIT nanoparticles without NIR irradiation. (B) Huh7 cell viabilities of ZSZI, ZSZIT and ZSZIT (pH = 6.0) under normoxia condition (80 µg/mL). (C) Huh7 cell viabilities of ZSZI, ZSZIT and ZSZIT (pH = 6.0) under hypoxia condition (80 µg/mL). (D) The fluorescence images of Huh7 cells cultured with different conditions (Blank control, ZSZIT + Dark, ZSZIT + NIR and ZSZIT + NIR under hypoxia) by DCFH-DA staining for ROS detection (80 µg/mL). (E) H_2_S detection within Huh7 cells after culturing with ZSZIT under normal and acidic conditions (80 µg/mL). (F) The fluorescence images of intracellular O_2_ in Huh7 cells incubated with PBS, ZSZIT in dark, with NIR irradiation, acidic condition in dark and with NIR irradiation (80 µg/mL). (G) Schematic diagram of mechanism of ZSZIT as H_2_S-sensitized PDT/chemotherapeutic synergistic therapeutic nanoplatform.

The cytotoxicity of as-prepared samples was evaluated in the absence of NIR laser using a Huh7 cell line. It is clear that ZSZI and ZSZIT do not present clear negative effect to cell viability at the concentration range of 0-90 μg/mL (Figure 4A). 7702 cell lines were also used to validate the cytotoxicity of nanoparticles, indicating no distinguishable toxicity to normal cells (Figure S15A). In addition, medium acidity was regulated to stimulate the tumor environment (pH = 6.0). Both ZSZI and ZSZIT exhibit modest toxicity compared with the blank control (Figure S15B). Since Zn^2+^ ions possess no clear toxicity to Huh7 cells either (Figure S16), the gentle toxicity observed may be potentially attributed to the release of H_2_S.

Subsequently, the *in vitro* antitumor effect of ZSZIT was evaluated systematically under normoxia and hypoxia conditions. With a normoxia culture condition, cells incubated with ZSZI exhibits clear decline of viability under 808 nm laser irradiation, which originates from singlet oxygen generation of ICG. ZSZIT-treated group exhibits significant cell killing effect, which can be attributed to the hypoxia-activated toxicity of TPZ enabled by the oxygen consumption during ROS induction by ICG. More importantly, when the pH of culture medium was decreased to 6, cells incubated with ZSZIT exhibits promoted inhibition to cancer cells comparing to than with a neutral pH, implying H_2_S, released in the acid, further amplify the killing effect of TPZ (Figure 4B). In comparison, the inhibition of ZSZI alone, by the ICG under NIR irradiation, is remarkably weakened under the hypoxia condition due to the insufficient oxygen supply. Comparing with the ZSZI-treated group (NIR irradiation) and ZSZIT-treated group (in dark), ZSZIT induces considerable cell killing effect due to TPZ agitated by the hypoxia during cell culture (Figure 4C and Figure S17). The acid-treated group also shows the significant cell killing effect due to the H_2_S released. Furthermore, AM/PI assay shows the similar apoptotic phenomena of all sample groups in comparison to the findings above (Figure S18). The intracellular ROS was further examined to illustrate the cascaded synergistic process within cells. Under a normoxia condition, ZSZIT under NIR irradiation induces considerable content of ROS within tumor cells, but in contrast its ROS induction is dramatically weakened under hypoxia condition (Figure 4D and Figure S19).

### Mechanisms

To uncover the functioning mechanism of ZSZIT in cancer cells, the intracellular H_2_S production was examined using WSP-1 probe. Cells incubated with ZSZIT at pH = 6.0 exhibit a clear green fluorescence, indicating the presence of H_2_S induced (Figure 4E). In comparison, no obvious green fluorescence is observed at a normal pH, as expected. In addition, the intracellular O_2_ presence was also examined. The NIR-triggered ROS induction consumes intracellular O_2_, and thus a red fluorescence presents in cells. Under an acid treatment, promoted red fluorescence is induced, indicating the intracellular H_2_S induces a sharp oxygen decline and more severe hypoxia condition (Figure 4F). The overall phenomenon is now clear. As demonstrated in Figure 4G, ICG delivered by ZSZIT produces ^1^O_2_ effectively under an 808 nm NIR irradiation, while consuming intracellular oxygen. Meanwhile, H_2_S is induced intracellularly due to the degradation of ZnS in the acidic condition, which could regulate a number of key biological functions. Herein, despite its inhibition effect to Huh7 cancer cells, H_2_S also downregulates the CAT activity 45, 46, enabling the cutting of transformation from H_2_O_2_ to O_2_ and promoted hypoxia condition (Figure S20). The toxicity of intracellular TPZ delivered by ZSZIT is agitated and activated by the severe hypoxia condition. In consequence, considerable inhibition to tumor cells is achieved due to the combined effects of intracellular ROS, H_2_S and activated TPZ enabled by ZSZIT particles.

### *In vivo* anti-cancer treatment

**Figure 5:**
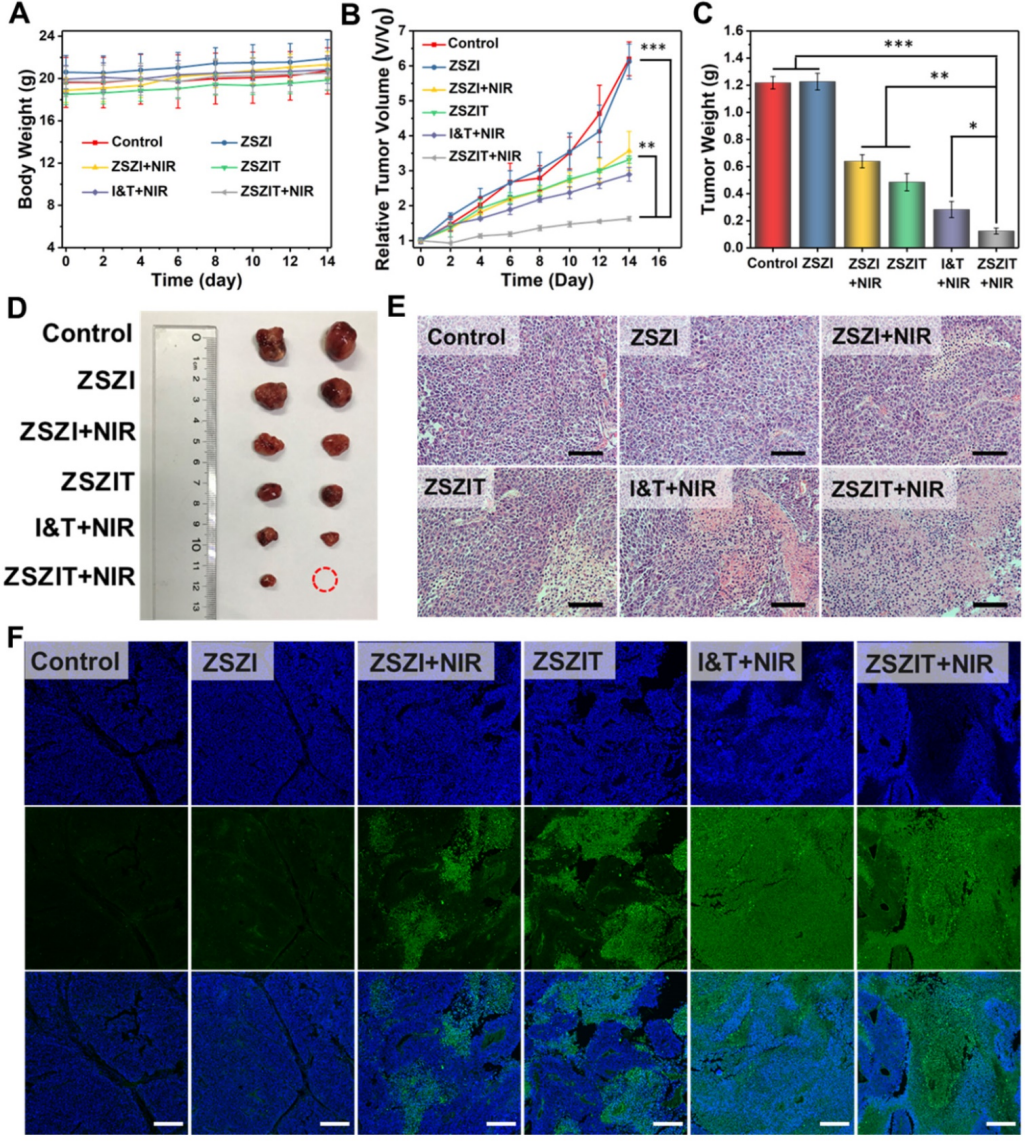
(A) Body weight and (B) tumor volume of mice in the following 2 weeks after receiving treatments. (C) Tumor weight and (D) representative photograph of the tumors collected from different groups of mice at day 14. (E) H&E stained tumor slices from different groups (Scale bar: 50 µm). (F) TUNEL stained tumor slices from different groups (scale bar: 250 µm).

The *in vivo* therapeutic effect of ZSZIT nanoplatform was further assessed using 6 groups of Huh7 tumors bearing male Balb/c nude mice. As shown in Figure 5A, body weight of mice presented no clear variation for all 6 groups, indicating negligible side effect. Tumor growth in Group 3 (injected with ZSZI and irradiated with NIR) was effectively inhibited by a certain magnitude comparing to Group 1 (control) and Group 2 (injected with ZSZI but without NIR), suggesting the NIR-triggered PDT effect. Tumor growth of mice in Group 4 (injected with ZSZIT only) was suppressed comparing to the control group due to the chemotherapeutic effect of TPZ in the hypoxic tumor environment. More importantly, Group 6 (injected with ZSZIT and irradiated with NIR) exhibited considerable enhanced tumor inhibition phenomenon compared with Group 3 and Group 4. In addition, tumor inhibition of Group 6 was found to be superior to the mice injected with free ICG and TPZ directly (Group 5), indicating that the H_2_S-sensitized PDT/chemotherapeutic nanoplatform effectively favors the tumor inhibition in an *in vivo* environment (Figure 5B). It is clear that tumors in Group 6 possess the lowest tumor weight and the smallest dimension, further verifying the most significant therapeutic effect of ZSZIT under 808 nm NIR irradiation than all other treatments (Figure 5C, 5D and Figure S21). The assay of HIF-1α expression, by immunohistochemistry staining, indicates that ZSZIT-treated group (with NIR) caused a severe hypoxia condition in the tumor tissue (Figure S22). Moreover, H&E analysis of tumor slices collected from each group suggests that the injection of ZSZIT after 808 nm NIR irradiation induced most severe damage to the tumor tissue due to its H_2_S-amplified synergistic therapeutic effect (Figure 5E). In comparison, ZSZI-treated group (Without NIR irradiation) was hardly affected compared with control group, verifying its negligible toxicity at a tissue level which agrees well to the findings of *in vitro* study. The TUNEL images confirmed the most significant apoptosis in ZSZIT-treated group (NIR irradiation), indicating its most efficient tumor inhibition (Figure 5F). More importantly, no clear sign indicates the side effects induced by ZSZIT to main organs (Figure S23 and S24), implying its systemic biosafety.

## Conclusion

In this work, a versatile nanoplatform, consisting core-shell ZnS@ZIF-8 nanoparticles incorporated with ICG and TPZ (ZSZIT), was designed and synthesized to enable H_2_S-sensitized chemo-/PDT synergistic therapy. Under an 808 nm NIR irradiation, ZSZIT induces ROS effectively while consuming the on-site oxygen. Meanwhile, in the acidic TME, ZIF-8 shell is decomposed, and ZnS cores are degraded to produce H_2_S gas *in situ*. The intracellular H_2_S does not only exhibit certain cytotoxicity, but also downregulates the expression of CAT, cutting the pathway of transformation from H_2_O_2_ to oxygen. The aggravated hypoxia activates TPZ molecules and induces severe cell killing effect. In consequence, considerable anticancer effect, both *in vitro* and *in vivo*, is achieved due to the combined effects of intracellular ROS, H_2_S and activated TPZ enabled by ZSZIT nanoparticles. This study has therefore offered a highly potential platform that enables gas-amplified cancer treatment with high efficacy.

## Supplementary Material

Supplementary figures.

## Figures and Tables

**Table 1:** Abbreviation of the samples

Sample abbreviation	Explanation
ZSZ	ZIF-8 coated ZnS: ZnS@ZIF-8
ZSZI	ZnS@ZIF-8 loaded with ICG
ZSZIT	ZnS@ZIF-8 loaded with ICG and TPZ
I&T	Mixture of free ICG and TPZ

## Abbreviations

TME: tumor microenvironment
ZSZ: ZnS@ZIF-8
ZSZI: ZnS@ZIF-8 loaded with ICG
ZSZIT: ZnS@ZIF-8 loaded with ICG and TPZ
SEM: scanning electron microscopy
TEM: transmission electron microscopy
XRD: X-ray diffraction
CCK-8: cell counting kit-8
PDT: photodynamic therapy
TPZ: tirapazamine
ICG: indocyanine green
ZIFs: zeolitic imidazolate frameworks
CAT: catalase
DPBF: 1,3-diphenylisobenzofuran
PVP: polyvinylpyrrolidone
PSS: poly (sodium 4-styrenesulfonate)
FTIR: fourier transform infrared spectroscopy
DI: deionized water
2-MIM: 2-methylimidazole
LC: loading capacity
EE: encapsulation efficiency
DCFH-DA: dichlorofluorescein diacetate
H&E: Histology examination
