# Supplementary material for: ZnS@ZIF-8 core-shell nanoparticles incorporated with ICG and TPZ to enable H2S-amplified synergistic therapy
Source: Theranostics. 2020 Jun 18;10(17):7671–82. doi: 10.7150/thno.45079 (PMC7359076; doi:10.7150/thno.45079)
Supplement: Supplementary file 1 — Supplementary figures. [file thnov10p7671s1.pdf]

Supporting Information

ZnS@ZIF-8 core-shell nanoparticles loaded with ICG/TPZ to enable H_2S-amplified synergistic therapy

Chao Fang, [1,+] Dong Cen,[2,+] Yifan Wang,[2] Yongjun Wu,[1] Xiujun Cai,[2] Xiang Li,[1,3] and Gaorong Han[1]*

1. State Key Laboratory of Silicon Materials, School of Materials Science and Engineering, Zhejiang University, Hangzhou 310027, P.R. China
2. Key Laboratory of Endoscopic Technique Research of Zhejiang Province, Sir Run Shaw Hospital, Zhejiang University, Hangzhou 310016, P. R. China
3. Hangzhou Global Scientific and Technological Innovation Center, Zhejiang University, Hangzhou, P.R. China
+Authors with equal contribution

*Corresponding authors: xiang.li@zju.edu.cn (XL)

Keywords: hydrogen sulfide, core-shell nanoparticles, indocyanine green, tirapazamine, synergetic therapy

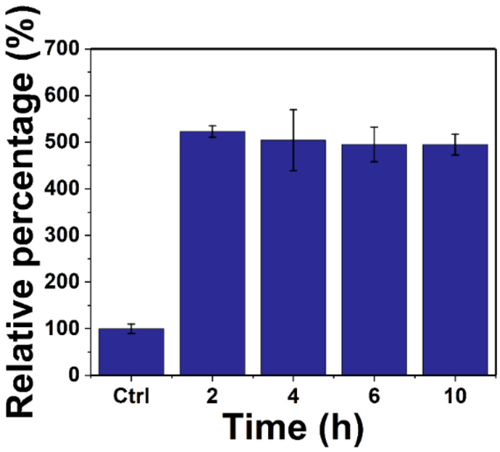

Figure S1. ICP analysis of Huh7 cells incubated with ZSZIT for different time (Control: without treatment, 100%).

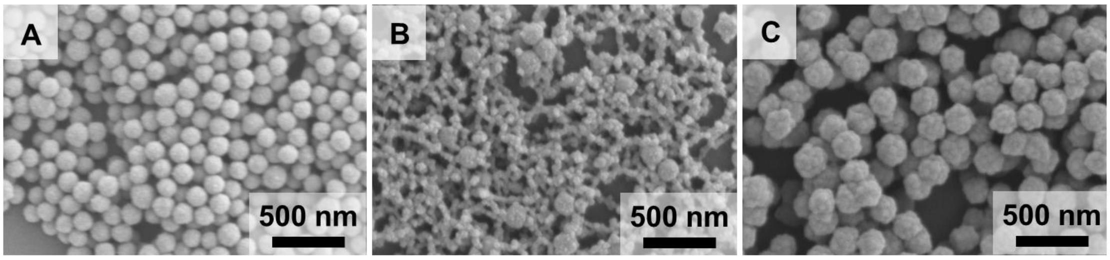

Figure S2. SEM images of (A) ZnS-PSS, ZIF-8 growth on ZnS nanoparticles in (B) aqueous solution and (C) methanol solution.

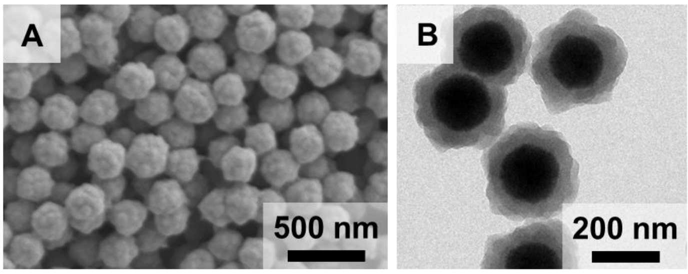

Figure S3. (A) SEM and (B) TEM images of ZSZ with the coating thickness of 50-60 nm.

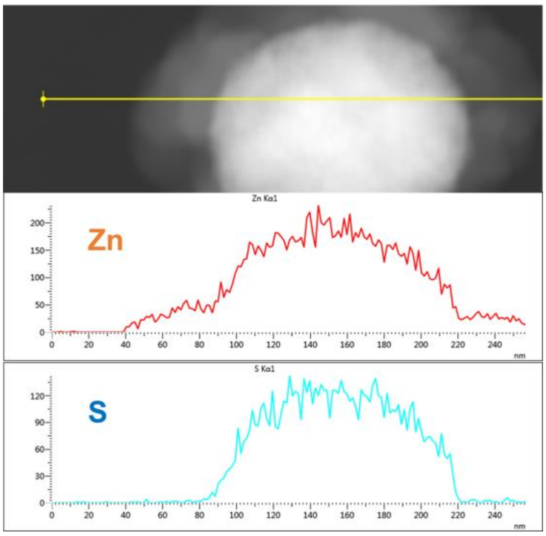

Figure S4. Corresponding EDS line-scan of ZnS@ZIF-8 nanoparticles.

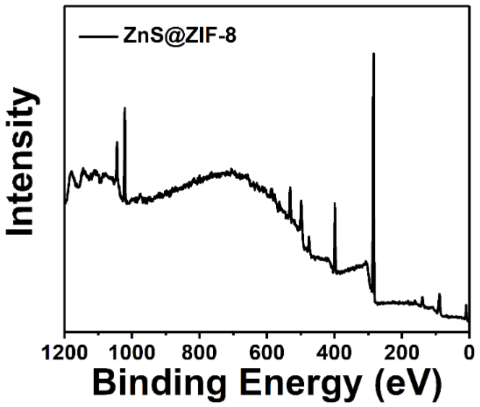

Figure S5. XPS spectra of ZnS@ZIF-8 nanoparticles.

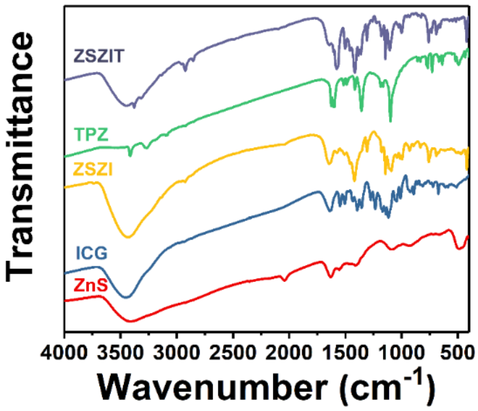

Figure S6. FT-IR spectra of ZnS, ICG, ZSZI, TPZ and ZSZIT.

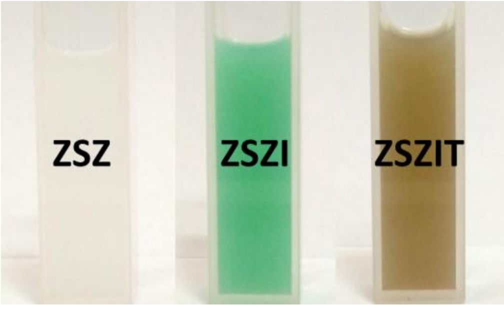

Figure S7. Photograph of methanol solutions containing ZSZ, ZSZI and ZSZIT.

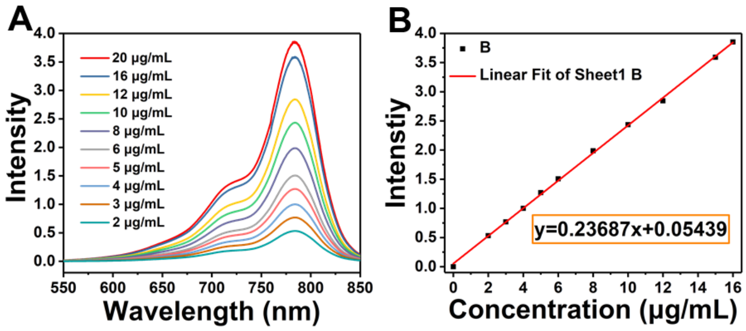

Figure S8. Standard curves of ICG concentration in methanol solution at the peak of 780 nm: (A) UV-vis absorbance spectra and (B) plotting curve of ICG methanol solution at different concentrations.

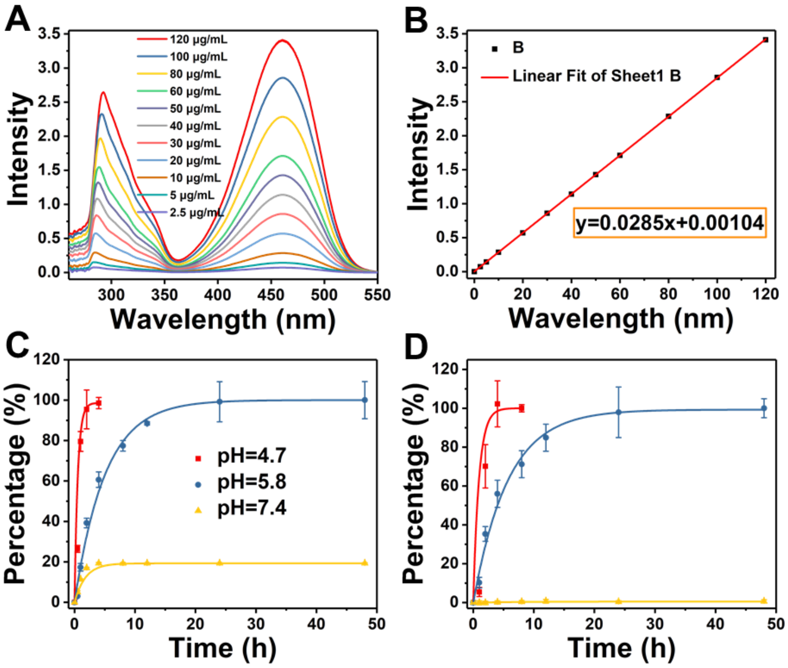

Figure S9. (A) UV-vis absorbance spectra and (B) plotting curve of TPZ aqueous solution at different concentrations; pH-triggered (C) TPZ and (D) ICG release behavior (500 μg/mL).

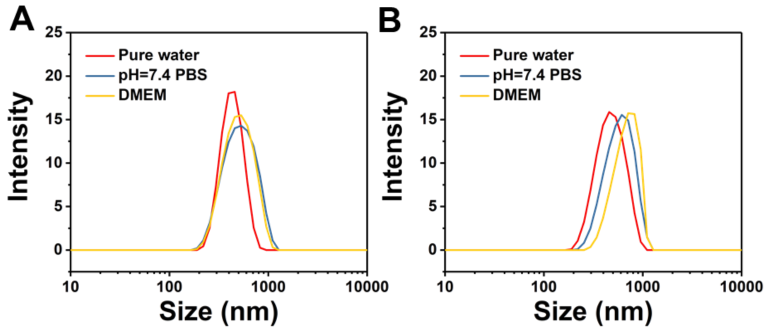

Figure S10. DLS results of ZSZIT nanoparticles suspended in different solutions including pure water, PBS and DMEM after (A) 0 h and (B) 12 h.

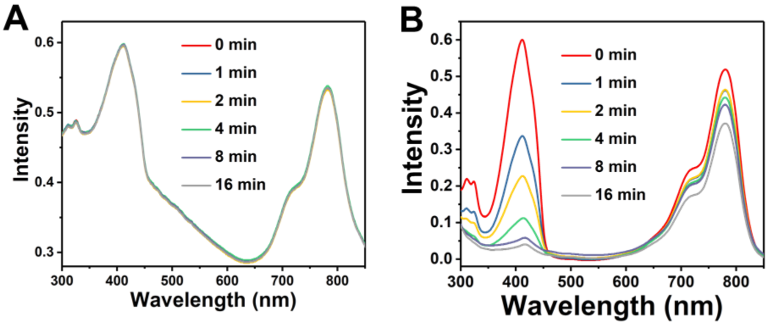

Figure S11. DPBF degradation in ZSZI solutions (A) without and (B) with 808 nm laser irradiation.

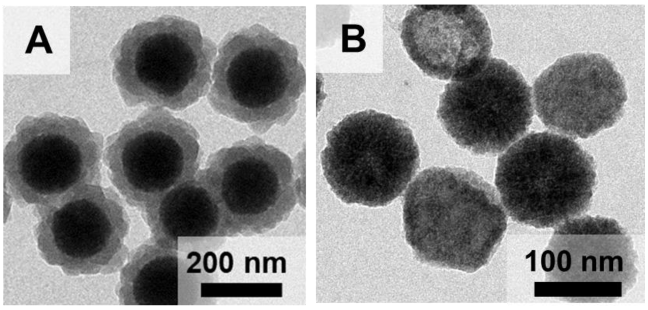

Figure S12. TEM images of ZSZI nanoparticles after immersion in solutions at (A) pH=7.4 and (B) pH=4.7 for 24 h.

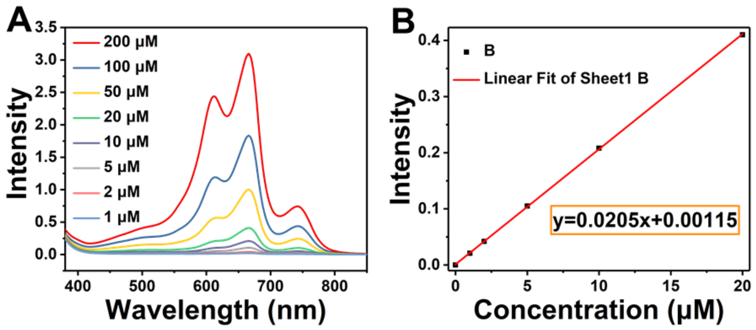

Figure S13. (A) UV-vis absorbance spectra and (B) plotting curve of methylene blue generated by Na_2S standard solution.

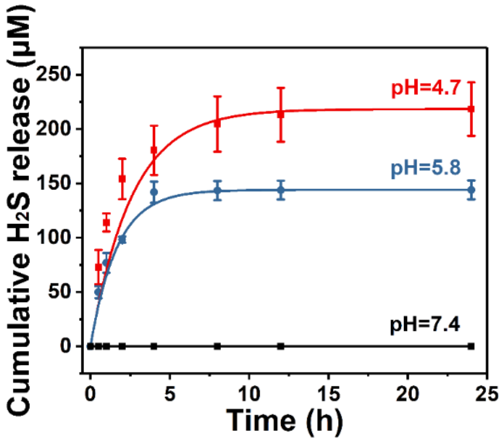

Figure S14. H_2S release profiles of ZnS nanoparticles in solutions with different pH (500 μg/mL).

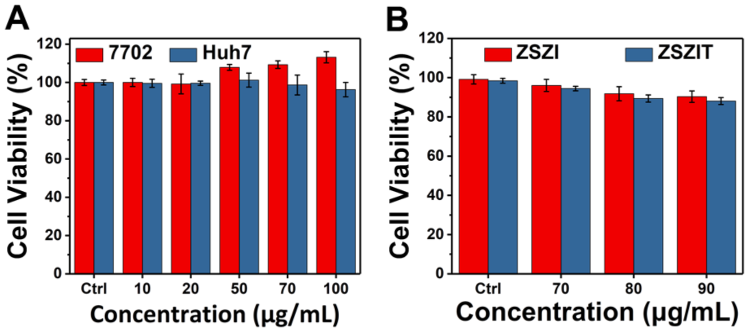

Figure S15. (A) Viabilities of 7702 and Huh7 cells cultured with ZSZI nanoparticles. (B) Viabilities of Huh7 cells cultured with ZSZI and ZSZIT at pH=6.0.

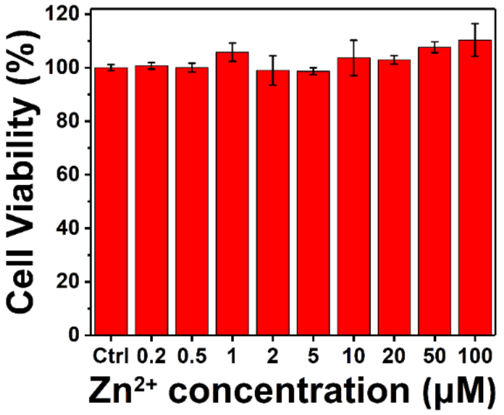

Figure S16. Huh7 cell viabilities with different concentrations of Zn^{2+} ions (0-100 μM).

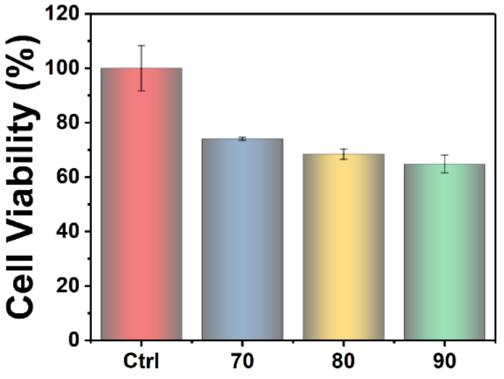

Figure S17. Huh7 cell viabilities of ZSZIT nanoparticles without NIR irradiation (Under hypoxia condition).

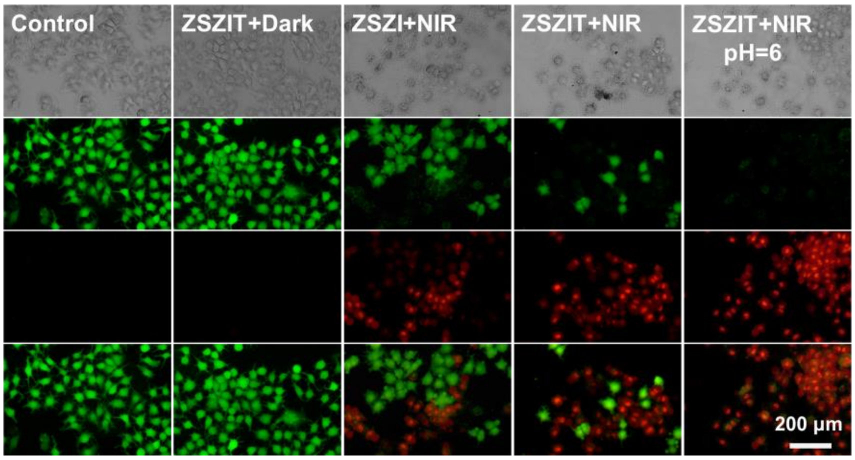

Figure S18. The bright field images, fluorescence images and merged fluorescence images of Huh7 cells incubated with PBS, ZSZIT+dark, ZSZI+NIR, ZSZIT+NIR and ZSZIT+NIR (pH=6) after Calcein AM and PI co-staining.

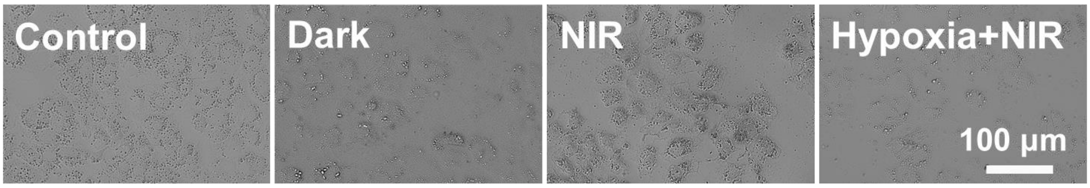

Figure S19. (a) The bright field images of Huh7 cells cultured with different conditions (Blank control, ZSZIT+Dark, ZSZIT+NIR and ZSZIT+NIR under hypoxia) by DCFH-DA staining for ROS detection.

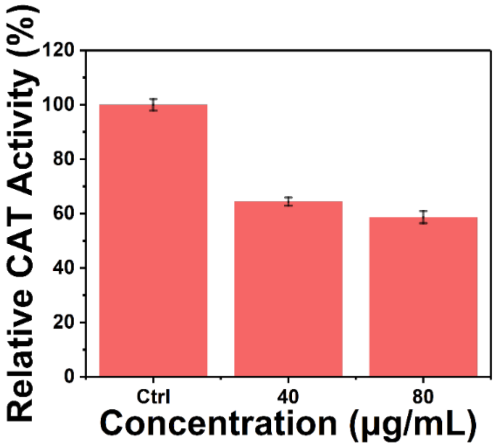

Figure S20. Effect of ZSZIT on CAT activity of Huh7 cells. CAT activities were determined by Catalase Assay Kit according to its protocol.

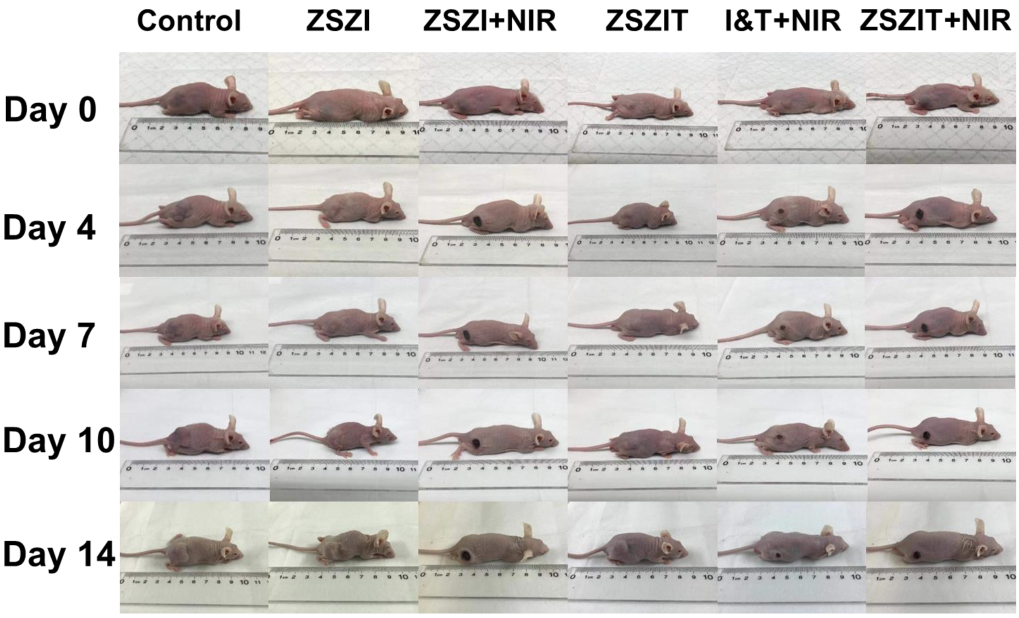

Figure S21. Representative photographs of mice from each group in the following 14 days after treatments (Day 0, 4, 7, 10 and 14).

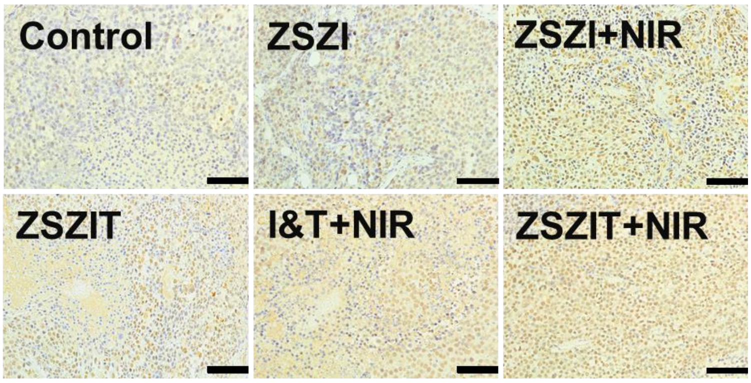

Figure S22. HIF-1α stained tumor slices from different groups (Scale bar: 100 μm.)

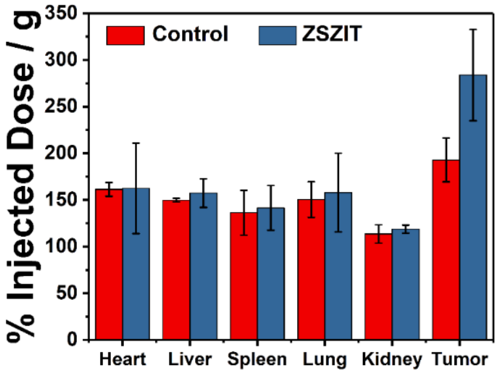

Figure S23. The biodistribution of Zn (% injected dose of Fe per gram of tissues) in main organs after one-day treatment.

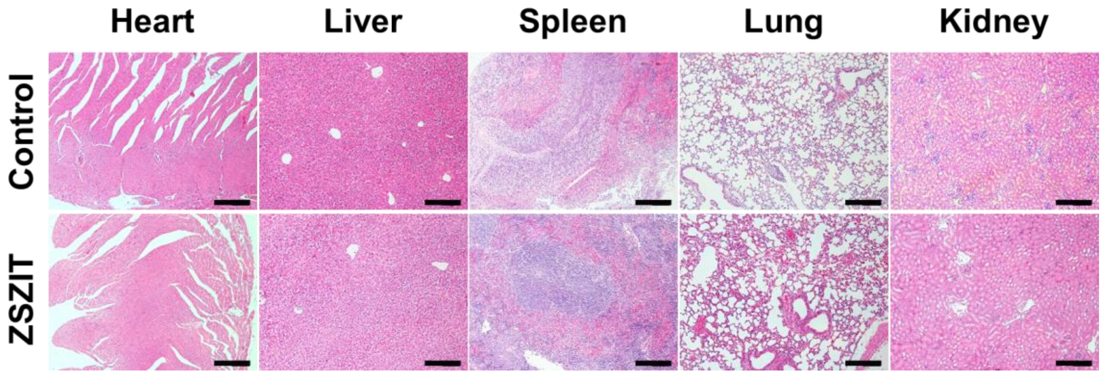

Figure S24. H&E stained images of main organs from mice treated with PBS and ZSZIT. Scale bar, 200 μm
